# Count highly-cited papers instead of papers with *h* citations: use normalized citation counts and compare “like with like”!

**DOI:** 10.1007/s11192-018-2682-1

**Published:** 2018-03-12

**Authors:** Lutz Bornmann, Loet Leydesdorff

**Affiliations:** 1 Division for Science and Innovation Studies, Administrative Headquarters of the Max Planck Society, Hofgartenstr. 8, 80539 Munich, Germany; 2 Amsterdam School of Communication Research (ASCoR), PO Box 15793, 1001 NG Amsterdam, The Netherlands

**Keywords:** *h*-Index, Bibliometric databases, Highly-cited papers

## Abstract

Teixeira da Silva and Dobránszki (Scientometrics. 10.1007/s11192-018-2680-3, 2018) describe practical problems in using the *h*-index for the purpose of research evaluation. For example, they discuss the *h*-index differences among the bibliometric databases. In this Letter to the Editor, we argue for abstaining from using the *h*-index. One can use normalized indicators instead.

Teixeira da Silva and Dobránszki (2018) describe practical problems in using the *h*-index for research evaluation purposes. (1) Teixeira da Silva and Dobránszki (2018) signal the problem of receiving different *h*-index values for the same researcher, if different (literature) databases—Scopus, Web of Science (WoS), ResearchGate (RG) or Google Scholar (GS)—are used as data sources. (2) They address the problem of generating correct publication profiles for researchers.

In this Letter to the Editor, we comment on these two points and argue for using counts of highly-cited papers as a better alternative to the *h*-index.

In the first part of their Letter to the Editor, the authors list well-known disadvantages of the *h*-index. However, two important disadvantages are not mentioned: (1) Waltman and Van Eck (2012) show that the *h*-index does not fulfil a property which is important in the application of indicators in research evaluations: “If two scientists achieve the same relative performance improvement, then their ranking relative to each other should remain unchanged” (p. 409). (2) Hirsch (2005) does not justify why publications and citations should be combined in the proposed way: “the number of papers with citation number > *h*” (p. 16569). Other ways of combining the numbers, such as using *h*^*2*^ or *h*/2 in the definition, are equally possible (Egghe 2006; Waltman and Van Eck 2012). Especially these two disadvantages question the use of the *h*-index in research evaluations.

## Different databases

Teixeira da Silva and Dobránszki (2018) assume that there exists a “true” value of the *h*-index that can be reached if the dataset were completely independent of the objectives of the database. In our opinion, such a “true” value does not exist; the *h*-value is database-dependent. Several bibliometric studies have pointed to (large) differences between the databases, which are mainly driven by different coverages of the literature leading to different citation counts for one and the same paper (e.g., Harzing and Alakangas 2015; Mongeon and Paul-Hus 2016).

The WoS, for example, does not claim to cover the complete set of publications, but a core selection. With a reference to Bradford’s (1934) Law, Garfield (1971) argued in favor of a core selection of journals representing the entire journal set. At the other extreme, GS collects information using web-spiders, including non-scholarly literature, pre-publications, and various versions of the same publication without clear selection criteria of quality. Scopus follows the WoS model, but includes more journals than WoS. RG is primarily a repository of preprints; the collection allows for the definition of a database-specific *h*-value.

Given these different objectives, the expectation is not that the *h*-index values are similar or even convergent across databases. From this perspective, Table 1 in Teixeira da Silva and Dobránszki (2018) teaches us that only eight of the 972 papers of the first author are in the *h*-core when using WoS. Only 25 of these 972 publications of the first author are listed in WoS; 49 of the 100 for the second author. It follows that the work of the first author is not scholarly oriented.

The conclusion drawn by these authors that WoS and Scopus do “not represent an accurate portrayal of the real publication status”, is not correct. The publication volume in terms of papers is different from the publication status of authors. The volumes represented in WoS and Scopus do not differ significantly from “reality”—as the authors claim—but these databases are based on professional criteria, while this is not the case for GS or RG. The claim that Scopus and WoS should not be used “until at least the 95th percentile of publications” is included, is based on misunderstanding the nature of these databases. Why should 95% of the football clubs play in the Champion’s League?

## Comparison of *h*-index values

The comparability of *h*-index values across the databases is to be distinguished from the comparability within each of the sources. The latter *h*-index values are only comparable after proper field-normalization. One cannot compare the *h*-index of a physicist with that from a physician or a historian. Field-delineations in the databases, however, are difficult (and largely unsolved; Leydesdorff and Bornmann 2016) and therefore comparisons of scholars in terms of *h*-index values remains error-prone. The problem is similar to that for other indicators, such as the journal impact factors.

For an author in the social sciences or the humanities, for example, reaching the level of highly-cited author according to their GS Citations public profiles (*h* > 100) is virtually impossible (see http://www.webometrics.info/en/node/58 for more details), whereas this is a realistic distinction in the life sciences. Similarly, a university with a large faculty of medicine cannot be compared with a university in which the focus is on technology, since the “citation potential” is low in the latter fields and high in the former (Garfield 1979; Moed 2010). But even when one compares “like with like” in the same database (Martin and Irvine 1983), differences may be indicated that are not based on differences in quality, but on differences in function, style, and objectives of specific publications. The inference to use these differences in article characteristics as indicators of differences among authors is further to be legitimated (Leydesdorff et al. 2016a).

## Normalization

Professional bibliometricians have frequently pointed out that normalized citations instead of bare citation counts should be used in research evaluations (Leydesdorff et al. 2016b). Using normalized indicators, the number of citations for a paper is standardized according to the expected citation rate of the corresponding field of publication. The importance of using normalized indicators has been highlighted, for example, in the Leiden Manifesto (Hicks et al. 2015).

A reasonable alternative to the *h*-index is to count the papers which belong to the top-cited papers in the corresponding fields (and not to count the number of papers in the *h* core). This method has been advocated by us (Bornmann and Marx 2014; Leydesdorff et al. 2011). Since researchers with different scientific ages are often to be compared in research evaluations, an age-normalized variant is the quotient of the number of highly-cited papers and the number of years since publishing one’s first paper.

The problem of receiving different *h*-index values depending on the database can perhaps be avoided by using normalized indicators. Normalized indicator values are more or less comparable across different data sources, since higher or lower citation counts are equalized by higher or lower expected values in the corresponding fields and publication years (Bornmann et al. 2016).

## Disambiguation

In the last part of their Letter to the Editor, Teixeira da Silva and Dobránszki (2018) elaborate on the problems with using automatically generated publication profiles for researchers. These profiles can be found in many databases, but should not be used without manual inspections. One cannot be sure that the profiles include all publications reliably (University of Waterloo Working Group on Bibliometrics 2016). According to Haustein and Larivière (2015) “disambiguation and cleaning author names and institutions is fundamental to computing meaningful bibliometric indicators used in research evaluation” (p. 127).

## Conclusion

We agree with Teixeira da Silva and Dobránszki (2018) that there are unsolved problems with using the *h*-index for research evaluation. However, the *h*-index is not a natural phenomenon: it contains necessarily a model of how to relate publications and citations as two very different things (Ye et al. 2017). The databases also contain models. The use of a model implies the generation of both error and information when making comparisons among model outcomes. Using the authors’ Table 1, for example, we have shown that the relatively low *h*-index values when using WoS do not lead to an argument against using this database, but can be considered as informative.

Normalization implies introduction of a third type of models. The *h*-index is then the wrong type of summary statistics. One should use non-parametric measures such as percentiles or quantiles instead (Hicks et al. 2015). The top-10% most-highly cited papers can be used for measuring excellence. In the case of rank-and-file authors, one is advised to use the Integrated Impact Index (*I3*) which normalizes quantile values across a distribution of citations (Leydesdorff and Bornmann 2012).
